# Involvement of the olfactory system in the induction of anti-fatigue effects by odorants

**DOI:** 10.1371/journal.pone.0195263

**Published:** 2018-03-29

**Authors:** Naoko Saito, Emi Yamano, Akira Ishii, Masaaki Tanaka, Junji Nakamura, Yasuyoshi Watanabe

**Affiliations:** 1 Kansei Science Research, Kao Corporation, Haga, Tochigi, Japan; 2 Health Metrics Development Team, RIKEN Compass to Healthy Life Research Complex Program, Kobe, Hyogo, Japan; 3 Department of Physiology, Osaka City University Graduate School of Medicine, Osaka, Japan; 4 Department of Sports Medicine, Osaka City University Graduate School of Medicine, Osaka, Japan; 5 RIKEN Center for Life Science Technologies, Kobe, Hyogo, Japan; University of Mississippi, UNITED STATES

## Abstract

Some components of the neural circuits underlying innate odor-evoked responses have recently been elucidated. Odor information detected by the olfactory receptors is transmitted from the olfactory bulb to the cortical amygdala, where physiological and emotional states such as attraction or avoidance are controlled. Thus, activation of specific olfactory receptors can elicit changes in physiological and/or psychological state. Here, we examined on the odorant Hex-Hex Mix, which has been reported to induce anti-fatigue effects. Fatigue is a prevalent condition that is often related to overwork and psychological stress. Various anti-fatigue treatments have been developed, including supplements and odorants. However, the mechanisms underlying the anti-fatigue effects of these substances are currently unclear. In the present study, we analyzed the involvement of the olfactory system in the mechanisms underlying this effect. We identified the human olfactory receptors activated by Hex-Hex Mix, and evaluated whether activation of these olfactory receptors by a newly developed odorant elicited a similar anti-fatigue effect to Hex-Hex Mix. We assessed anti-fatigue effects with behavioral tests, and 17 healthy males performed the 2-back test as a fatigue-inducing task with or without exposure to the new odorant. Immediately before and after the task, participants performed a cognitive task to evaluate their level of mental fatigue. We found that the difference value of the correct response rate on the cognitive task in the evaluation session was significantly different between in the odorant condition and in the without-odorant condition during the fatigue-inducing session suggesting that the new odorant may improve performance in the fatigue-inducing condition. The results indicated that the new odorant activates the same olfactory receptors as Hex-Hex Mix, which has been reported to induce anti-fatigue effects. Our findings suggest that the olfactory receptors in the olfactory system may be involved in the attenuation of fatigue.

## Introduction

Odorants are recognized by olfactory receptors (ORs) expressed on the olfactory sensory neurons in the olfactory epithelium. Mammalian ORs are seven transmembrane G protein coupled receptors [1]. In a previous study, it was reported that 396 OR genes in humans, and 1035 OR genes in the mouse constitute one of the largest gene families in the mammalian genome [2]. In the mammalian olfactory system, an odorant molecule is recognized by a number of ORs, and an OR can recognize multiple odorant molecules. This mechanism enables us to discriminate hundreds of thousands of odorants [3,4]. Thus, the perceptual qualities of an odor are determined by the OR code that is activated.

The axons of the OR neurons expressing the same OR converge on the glomeruli in the olfactory bulb. The odor information detected by ORs in the olfactory epithelium is then converted to a topographical map of the pattern of activated glomeruli in the olfactory bulb [5]. Previous findings suggest that the topographic odor information in the olfactory bulb induces perceptual and behavioral properties [6].

A number of odorants have been found to affect emotional state and physiological function in mammals. In addition, studies with anosmic mice produced by olfactory bulbectomy or intranasal zinc treatment, have reported that some of these effects are mediated by olfactory systems [7,8,9]. Regarding emotional state, it has been found that 4-Hydroxy-3-methoxybenzaldehyde (vanillin) attenuated depressive symptoms in a rat model of chronic depression [7], whereas *Citrus sinensis* (sweet orange) essential oil induced anxiolytic effects in rats in the elevated plus-maze [10] and 4-(1-methylethenyl)-1-cyclohexene-1-carboxaldehyde (*l*-perillaldehyde) exhibited antidepressant-like effects in stress-induced depression-like model mice via olfactory nervous system function [8]. Regarding physiological function, terpinyl acetate and phenethyl alcohol was found to increase sleep time in mice [9], whereas *Rosa alba* (rose) essential oil prevented skin barrier disruption in rats and humans [11], and *Cananga odorata* (Ylang-Ylang) essential oil decreased blood pressure in humans [12]. Thus, odorants may provide a useful tool for treating a range of conditions in humans.

Fatigue is prevalent in many contemporary societies, and is often related to overwork and/or various types of psychological stress. Approximately 20%–30% of the general population in European countries and the United States experience substantial fatigue [13–17]. In Japan, more than half of the adult population reports experiencing fatigue [18]. Fatigue is defined as a decline in the ability to perform (or a reduction in the efficiency of performance) of mental and/or physical activities caused by excessive mental or physical activity or disease, and is often accompanied by a sense of discomfort, a desire to rest, and a decline in motivation [19]. In a previous study, fatigue was reported to increase the likelihood of workplace accidents, morbidity, and mortality [20,21]. Thus, the development of anti-fatigue treatments may have a range of benefits. Recent studies have reported that (-)-epigallocatechin gallate [22], chicken essence [23] and 2,3 dimethoxy-5 methyl-6-decaprenyl benzoquinone (coenzyme Q10) [24] induced anti-fatigue effects. Furthermore, some odorants, such as *Lavendula angustifolia* (Lavender) essential oil [25] and Hex-Hex Mix [26–28], have also been reported to exhibit anti-fatigue effects.

Hex-Hex Mix is a mixture of *cis*-3-hexenol and *trans*-2-hexenal, which gives off the odor of fresh green leaves and has verified anti-fatigue effects [26–28]. Exposure to Hex-Hex Mix has been found to prevent fatigue-related delay of reaction times in a range of tasks, including the simple visual discrimination task and the advanced trail making test (ATMT) [29]. In addition, the height of wave A, one of the parameters of the autonomic function assessed by acceleration plethysmography (APG) was found to be decreased after performing ATMT with exposure to Hex-Hex Mix [27]. This anti-fatigue effect has been shown in both monkey and human studies [26,27]. In addition Hex-Hex Mix has been shown to attenuate fatigue induced by forced swimming in rats [28]. A study using positron emission tomography (PET) reported that regional cerebral blood flow (rCBF) in the anterior cingulate gyrus was increased during exposure to Hex-Hex Mix in monkeys [26]. This finding suggests that Hex-Hex Mix may influence neural activity in the anterior cingulate gyrus, which is involved in autonomic nerve function. Thus, the reported fatigue-mitigation effects of Hex-Hex Mix may operate via the autonomic nervous system, potentially exhibiting a healing effect on the sympathetic nervous system [26,27]. However, the detailed physiological mechanisms by which Hex-Hex Mix attenuates fatigue remain unclear.

Recently, the OR codes of various odorants have been identified using a heterologous expression system [30,31] to understand the combinatorial nature of olfactory coding. OR7D4 has been identified as a receptor for androstenone, and its function is related to the human perception of androstenone odor [32]. In the present study, we focused on ORs that are activated by Hex-Hex Mix, to clarify the relationship between the olfactory system and the anti-fatigue effects of odorants in humans. We hypothesized that if Hex-Hex Mix receptors are involved in the induction of physiological functions (e.g., anti-fatigue effects), other ligands of Hex-Hex Mix receptors would be expected to induce the same physiological effect. To test this hypothesis, we developed a new odorant called MCMP, a mixture of methyl ß-naphthyl ketone, 2-Methyl-5-(1-methylethenyl)-2-cyclohexenone (*l*-carvone), 1,2-dimethoxy-4-(prop-1-en-1-yl)benzene (methyl isoeugenol) and phenylethyl acetate that has a honey-like floral odor by analyzing the activation of Hex-Hex Mix receptors and assessed its anti-fatigue effects with behavioral tests in humans.

In the present study, we evaluated fatigue levels using reaction time and the correct response rate in Task B of the modified Stroop color-word test in reference to previous studies that investigated anti-fatigue or fatigue recovery effects from fatigue [23, 33]. This task was based on the Stroop test [34], and consisted of modified Stroop trials (in which the color of a traffic light did not match the meaning of a character) and non-Stroop trials (in which the color of a traffic light matched the meaning of a character) [33].

## Materials and methods

### Cloning

All of the human OR genes were amplified from human genomic DNA (Promega, Madison, USA) using KOD FX (TOYOBO, Osaka, Japan) with sequence information from NCBI. Each forward primer included the EcoR I sequence followed by 25 to 40 base pairs of N-terminal sequence of each OR. Each reverse primer included the Spe I sequence followed by 25 to 40 base pairs of C-terminal sequence of each OR. The amplified OR genes and pME18S vectors containing a FLAG tag and the first 20 residues of bovine rhodopsin followed by EcoR I and Spe I sequence were restricted by EcoR I and Spe I(TAKARA BIO INC. Shiga, Japan) and ligated using DNA Ligation Kit (TAKARA). The sequences of cloned ORs were verified by sequencing. Some of the genes included several single nucleotide polymorphisms (SNPs) with sequence information from NCBI dbSNP (build141; http://www.ncbi.nlm.nih.gov/SNP/). Human receptor-transporting protein 1 short (RTP1S) gene was amplified from the human RTP1 gene (GE Healthcare UK Ltd., Buckinghamshire, England) using KOD FX (TOYOBO) with sequence information from NCBI. Forward and reverse primer included EcoR I and Xho I sequence followed by N-terminal or C-terminal sequence of RTP1S, respectively. The amplified RTP1S gene and pME18S vector were restricted by EcoR I and Xho I (TAKARA) and ligated using DNA Ligation Kit (TAKARA). The sequences of cloned RTP1S were verified by sequencing.

### Luciferase assay

The firefly luciferase vector driven by the cAMP response element CRE/luc2P-pGL4.29 (Promega) was used to measure receptor activity. A *Renilla* luciferase vector driven by the thymidine kinase promoter TK/Rluc-pGL4.74 (Promega) was used for internal control of cell viability and transfection efficiency. An RTP1S pME18S vector was used to improve receptor transfer to the cell membrane. 75 ng of a FLAG-Rho-tagged OR pME18S vector, 30 ng of CRE/luc2P-pGL4.29, 30 ng of TK/Rluc-pGL4.74 and 30 ng of RTP1S pME18S vector were transfected to HEK293 cells (provided by Prof. Touhara at The University of Tokyo) using Lipofectamine 2000 (Thermo Fisher Scientific, Waltham, USA) in poly-D lysine-coated 96-well plates (Corning, Corning, USA), and incubated at 37°C in 5% CO_2_. For receptor screening for the odorant mixture, MCMP, 384 well plates were used, and the gene amounts used in the assay were adjusted to the area ratio with 96 well plates. Approximately 24 hours later, the medium was removed and the transfected cells were treated with odorant solution of an appropriate concentration in Dulbeccos Modified Eagle Medium (DMEM) without fetal bovine serum (FBS). The 96 or 384 well plates were sealed and incubated at 37°C for 4 hours. The luciferase reporter gene activities were measured with a Dual-Glo Luciferase Assay System (Promega) and the luminescence was measured with a Centro LB960 plate reader (Berthold Technologies, Bad Wildbad, Germany) or Ensight multimode plate reader (PerkinElmer, Waltham, USA). Fold increases were calculated as Luc(S) divided by Luc(NS), where Luc(S) was the luminescence intensity of firefly luciferase divided by the luminescence intensity of *Renilla* luciferase of a certain odorant-stimulated well, and Luc(NS) was the luminescence intensity of firefly luciferase divided by the luminescence intensity of *Renilla* luciferase of a certain non-stimulated well. For the identification of receptors that responded to Hex-Hex Mix, we evaluated the response of all ORs to Hex-Hex Mix, and selected candidate receptors that showed more than two fold increases. For MCMP, we conducted the receptor screening twice, and selected candidate receptors that exhibited more than two fold increases in both assays in addition to the identified Hex-Hex Mix receptors. We evaluated the dose-dependent response of candidate ORs three to six times, and identified the Hex-Hex Mix or MCMP receptors that exhibited significant responses against vector control. The odorants used in these assays were Hex-Hex Mix, which was an equal molar mixture of *cis*-3-hexenol (Merck, Darmstadt Germany) and *trans*-2-hexenal (Merck), and MCMP, which was an equal molar mixture of methyl ß-naphthyl ketone (Merck), *l*-carvone (Wako, Osaka, Japan), methyl isoeugenol (Merck), and phenylethyl acetate (Merck).

### Participants

Seventeen healthy male volunteers (aged 33.8 ± 11.7 years [mean ± SD]) with no abnormalities in their sense of smell were enrolled in this study. Current smokers, participants with a history of mental or brain disorders, and participants taking chronic medications affecting the central nervous system were also excluded. All participants provided written informed consent before participation. The study was approved by the Ethics Committee of Osaka City University and was conducted in accordance with the principles of the Declaration of Helsinki.

### Study design and procedures

**Fig 1:**
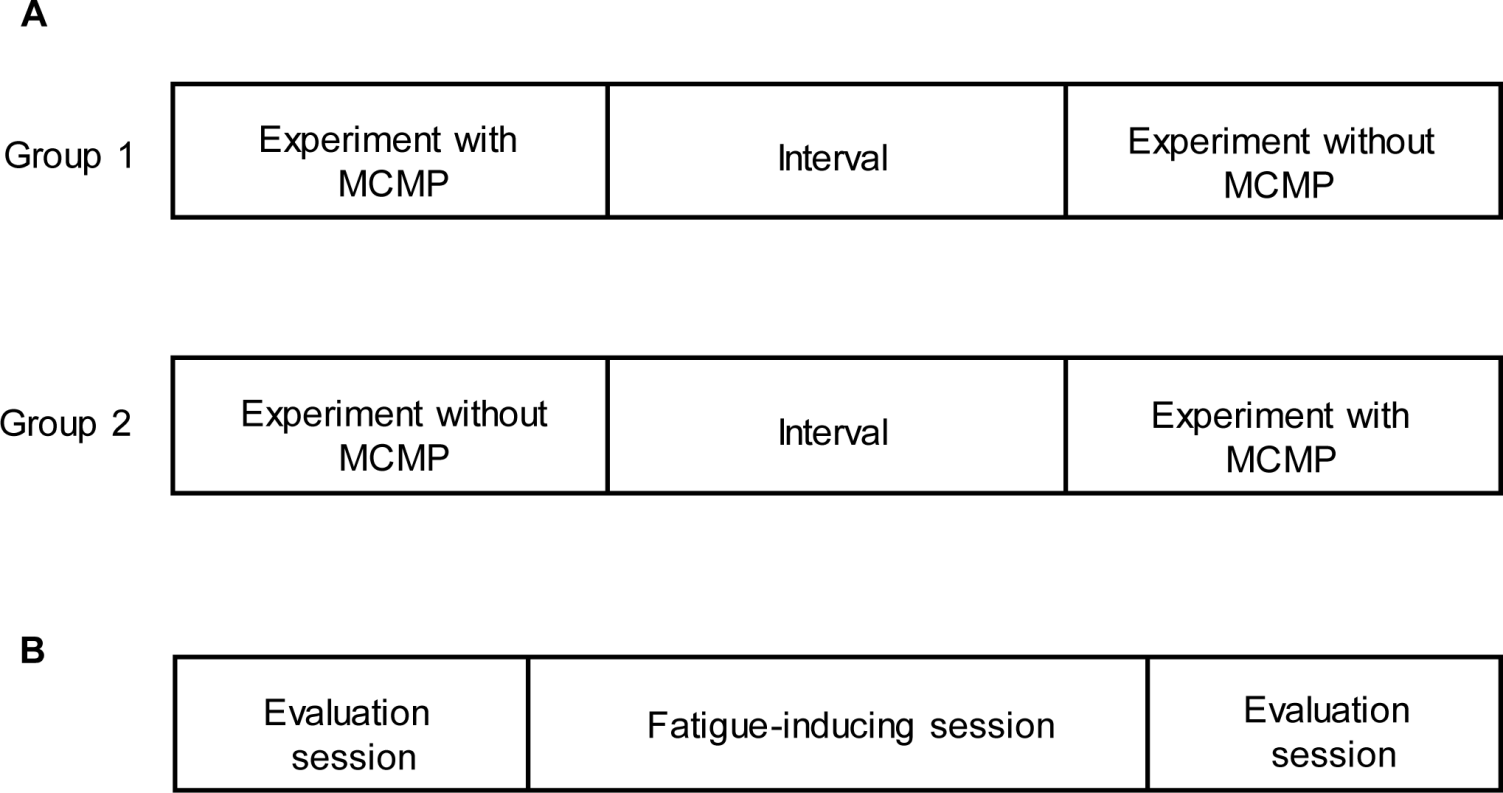
**Study design (A) and its procedure (B).** Participants were randomly assigned to one of two groups, and performed fatigue-inducing tasks with or without MCMP on successive days. Each study consisted of 40 min mental fatigue-inducing tasks (fatigue-inducing sessions) and two evaluation sessions (evaluation sessions). Each evaluation session was performed before and after each fatigue-inducing session.

The study consisted of two trials: a fatigue-inducing task with or without an MCMP condition. After enrollment, participants were randomly assigned to two groups in a crossover design to begin the study with or without MCMP during the fatigue-inducing task session (Fig 1A).

On each trial, participants performed the fatigue-inducing task session and two evaluation sessions just before and after the task session (Fig 1B). During the fatigue-inducing session, participants performed the 2-back test for 40 min with or without MCMP. During the evaluation session, participants performed a cognitive task for 9 min to assess the level of fatigue and rated their subjective level of fatigue using a 100-mm visual analogue scale (VAS) from 0 (minimum fatigue) to 100 (maximum fatigue). Autonomic nerve activity was also measured using accelerated plethysmography (APG) for 1 min. For 1 day before each visit, participants refrained from drinking caffeinated beverages and only ingested water during each trial. The study was conducted in a quiet temperature- and humidity-controlled environment.

### Stimulus presentation methods

To present the odorant, we used a diffuser (Ryohin Keikaku, Co., Ltd., Japan) and a plastic spray bottle (System Shine Service, Co., Ltd., Japan, 250 ml). The concentration of the MCMP solution was 0.02% for the diffuser and 0.06% for spray bottle, diluted with water. The diffuser was used during fatigue-inducing session for 40 min. In addition, mist was sprayed with the spray bottle twice every 5 min in the corner of the testing cubicle. In the control condition, only water was applied with the diffuser and plastic spray bottle.

The diffuser was placed on a fixed desk at the back of the testing cubicle, out of the participants’ sight. To maintain a uniform concentration of odorant throughout the trial, the diffuser was turned on 20 min before the fatigue-inducing session. Odor intensity was above the subjective detection threshold and of approximately equivalent strength for each trial, as assessed by an independent party. The cubicles for the fatigue-inducing session were 6.3 m long × 5.7 m wide × 2.5 m high, with identical insulation conditions (room temperature, 23–24°C; humidity, 35–45%). The stimulus was presented continuously for 40 min (fatigue-inducing session). Participants were instructed to breathe freely during the session. Throughout the fatigue-inducing sessions, tests with or without MCMP (control condition) were conducted under the same environmental conditions.

### Fatigue-inducing task

In the fatigue-inducing task, participants performed the 2-back test [35] for 40 min [36]. Four types of letters were continually presented in white on a black background on the display of a laptop computer, one at a time, every 3 s. The letters were 30 mm × 30 mm in size. During the test, participants were instructed to judge whether the target letter presented at the center of the screen was the same as the one that had appeared two presentations earlier. If the target letter was the same, they were to press the right button with their right middle finger, whereas if it did was not, they were to press the left button. Participants were instructed to perform the task as quickly and as accurately as possible. The result of each 2-back test (correct response or error) was continually presented on the display of the laptop computer.

### Cognitive task

The cognitive task presentation consisted of traffic lights (placed on a character corresponding to the words “blue” or “red” in Japanese) shown on the display of a laptop computer. Participants performed Task A (simple color reaction test) for 3 min and Task B (modified Stroop color-word test) for 6 min. In Task A, participants were instructed to press the right button with their right middle finger if the blue traffic light was presented (placed on the character corresponding to blue in Japanese). If the red traffic light was presented (placed on the character corresponding to red in Japanese), participants were instructed to press the left button with their right index finger. Task A was conducted to confirm that the colors could be distinguished and reported properly. In Task B, participants were instructed to judge whether the target character presented at the center of a traffic light was blue or red. If the character meaning blue in Japanese was presented, regardless of the color of the traffic light, participants were instructed to press the right button with their right middle finger; otherwise, they were instructed to press the left button with their right index finger. In these tasks, each trial was presented 100 ms after pressing either of the buttons. During the task period, blue or red trials were presented randomly, and the probability of occurrence of each color and type of sign was equal. In Task B, the modified Stroop trials (in which the color of the traffic light did not match the character) and non-Stroop trials (in which the color of the traffic light matched the character) [33] occurred an equal number of times. This task is based on the Stroop test [34], a widely-used experimental paradigm in research settings. In this test, participants are asked to name the color of visually-presented characters, either in a congruent or an incongruent condition. Strong interference between word reading and color naming is known as the Stroop interference effect, which occurs when the presented noun is a color or name displayed visually using a different color [33]. This paradigm has been used to demonstrate the limited capacity of the human attentional system in the selection of processing centers appropriate for job execution.

During the task period of blue or red trials, participants were instructed to press the right or left button as quickly and as accurately as possible. The result of each cognitive task (correct response or error) was continuously presented on the display of the laptop computer. The correct response rate was calculated from the number of correct responses in all trials in Task A and B, respectively.

In the present study, we focused on the reaction time and correct response rate of Task B in reference to previous fatigue-related studies [23, 33].

### Accelerated plethysmography (APG)

APG has been used for the evaluation of autonomic nerve activity [37–39]. In the present study, APG was performed using a pulsimeter (Artett, U-Medica, Osaka, Japan) with the sensor positioned on the tip of the ventral side of the index finger. Photoplethysmography was used to measure changes in the absorption of light by hemoglobin, which is related to blood flow volume, and the second derivative of the photoplethysmographic waveform was calculated. This is known as the APG waveform. Participants underwent APG while sitting quietly with their eyes closed for 1 min. The sensor output of the pulsimeter was preprocessed using a second-order analogue low-pass filter with a 23 Hz cut-off frequency. Data were recorded (3.3 volts to 10 bits) using an analogue-to-digital converter and a real-time sampling rate of 1,000 samples per second. These digital data were processed with a 67th order, finite impulse-response filter using a Hanning window. Detected peak times were interpolated in the sub-millisecond order. Frequency analyses for pulse-interval variation were analyzed using fast Fourier transformation. The resolution ability for the power spectrum was 0.001 Hz. For the frequency analyses, the total power was calculated as the power within a frequency range of 0–0.4 Hz, the low-frequency component power (LF) was calculated as the power within a frequency range of 0.04–0.15 Hz, and the high-frequency component power (HF) was calculated as that within a frequency range of 0.15–0.4 Hz. The average power densities in these frequency bands were log-transformed (ln) for normalization. The HF is vagally mediated [40–43], whereas LF originates from a variety of sympathetic and vagal mechanisms [40–43]. The LF/HF ratio is considered to represent sympathetic activity [43].

### Subjective scaling

Subjective scaling was performed to evaluate participants’ subjective levels of fatigue. Participants were asked to rate their subjective level of fatigue on a VAS from 0 (minimum) to 100 (maximum).

### Statistical analyses

Values are presented as mean ± SE unless otherwise stated. Two-way analysis of variance (ANOVA) with post-hoc Fisher’s lest significant difference (LSD) test was performed to identify ORs. Two-way ANOVA with repeated measures was performed to assess the effect of condition and time point. Paired t-tests with Bonferroni correction was used to compare the subjective level of mental fatigue, correct response rate, and reaction time of Task B across different time points in two conditions. Paired t-tests and Student’s t-tests were used to evaluate significant differences between the two conditions. All p-values were two-tailed, and values less than 0.05 were considered statistically significant. Statistical analyses were performed using SPSS for Windows version 22.0 (IBM, Armonk, NY) and GraphPad Prism (GraphPad Software, Inc, La Jolla, USA).

## Results

### Identification of ORs for Hex-Hex Mix

**Fig 2:**
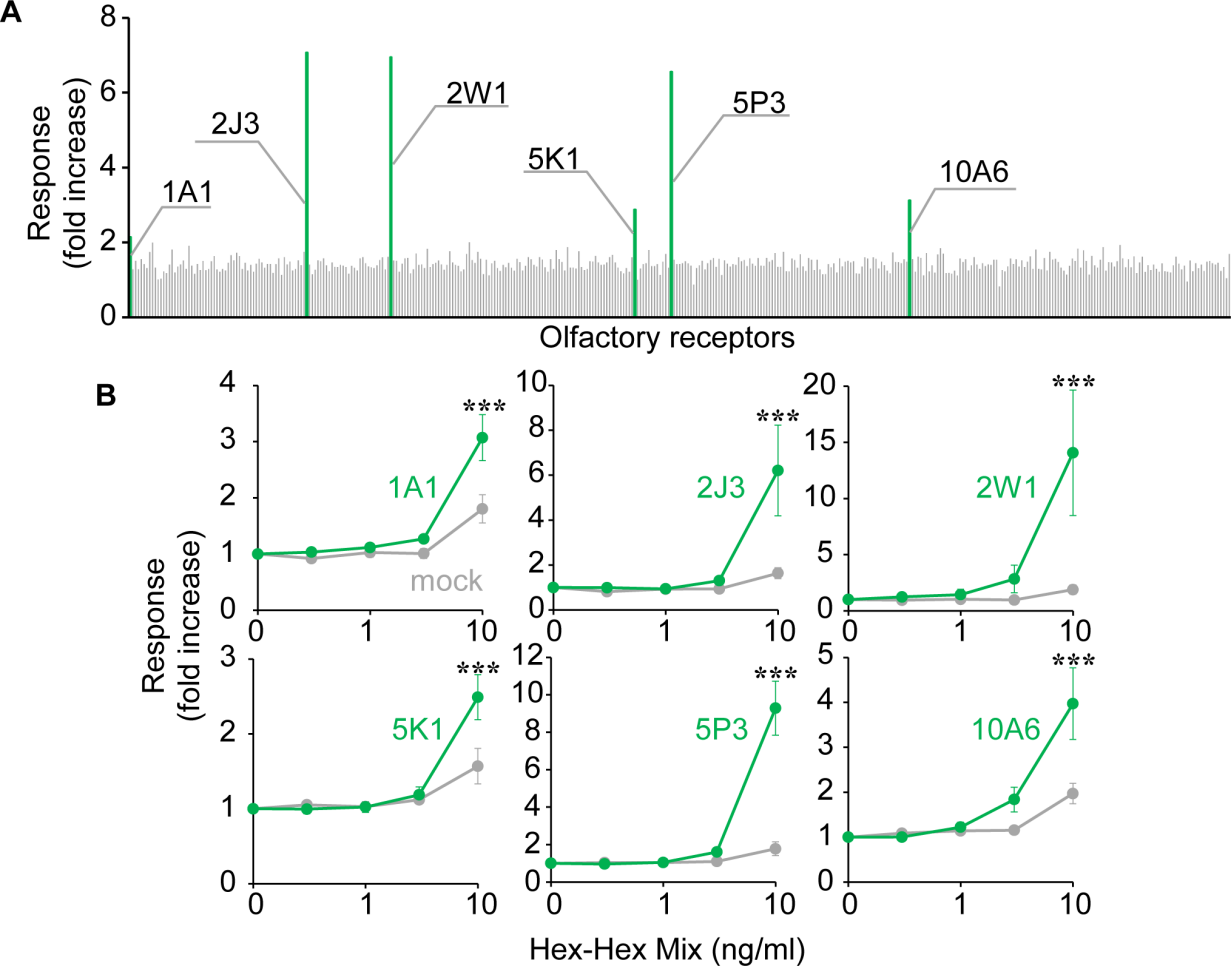
Screening of Hex-Hex Mix receptors from the human OR repertoire. (A) 392 human ORs were stimulated with 10 ng/ml Hex-Hex Mix comprised 50 μM cis-3-hexanol and 50 μM trans-2 hexenal, respectively, and luciferase assay was performed. Fold increases were calculated as mentioned in the Materials and Methods section. (B) Dose response curves of each OR. The HEK293 cells transfected with each OR were stimulated by 0.3 to 10 ng/ml of Hex-Hex Mix and luciferase assay was performed. Fold increases are shown as means and SE (n = 3–6). *p < 0.05, **p < 0.01, ***p < 0.001, Fisher's LSD post-test.

To identify the ORs activated by the anti-fatigue odorant Hex-Hex Mix, the entire range of human ORs were stimulated with 10 ng/ml Hex-Hex Mix, while we evaluated the increase in intracellular cAMP caused by OR activation with a luciferase assay system. Two-way ANOVA with post-hoc LSD was performed to compare the fold increases cross the particular receptor versus the mock transfection, concentration of Hex-Hex Mix and receptor × concentration interaction. There were main effects of the particular receptor versus the mock transfection (OR1A1, F [1, 50] = 11.73, p < 0.01; OR2J3, F [1, 40] = 6.294, p < 0.05; OR2W1, F [1, 20] = 6.541, p < 0.05; OR5K1, F [1, 20] = 4.900, p < 0.05; OR5P3, F [1, 20] = 28.20, p < 0.001; OR10A6, F [1, 40] = 9.141, p < 0.01) and concentration of Hex-Hex Mix (OR1A1, F [4, 50] = 30.40, p < 0.001; OR2J3, F [4, 40] = 8.259, p < 0.001; OR2W1, F [4, 20] = 5.419, p < 0.01; OR5K1, F [4, 20] = 22.29, p < 0.001; OR5P3, F [4, 20] = 35.29, p < 0.001; OR10A6, F [4, 40] = 17.24, p < 0.001). Each receptor × concentration interaction showed six ORs (OR1A1, OR2J3, OR2W1, OR5K1, OR5P3 and OR10A6), and exhibited responses to Hex-Hex Mix that were significantly above control levels in dose response curves ([OR1A1, F [4, 50] = 5.341, p < 0.01; OR2J3, F [4, 40] = 4.727, p < 0.01; OR2W1, F [4, 20] = 4.078, p < 0.05; OR5K1, F [4, 20] = 4.893, p < 0.01; OR5P3, F [4, 20] = 24.57, p < 0.001; OR10A6, F [4, 40] = 4.836, p < 0.01). (Fig 2 and S1 Table).

### Developing a new odorant for activating Hex-Hex Mix receptors

**Fig 3:**
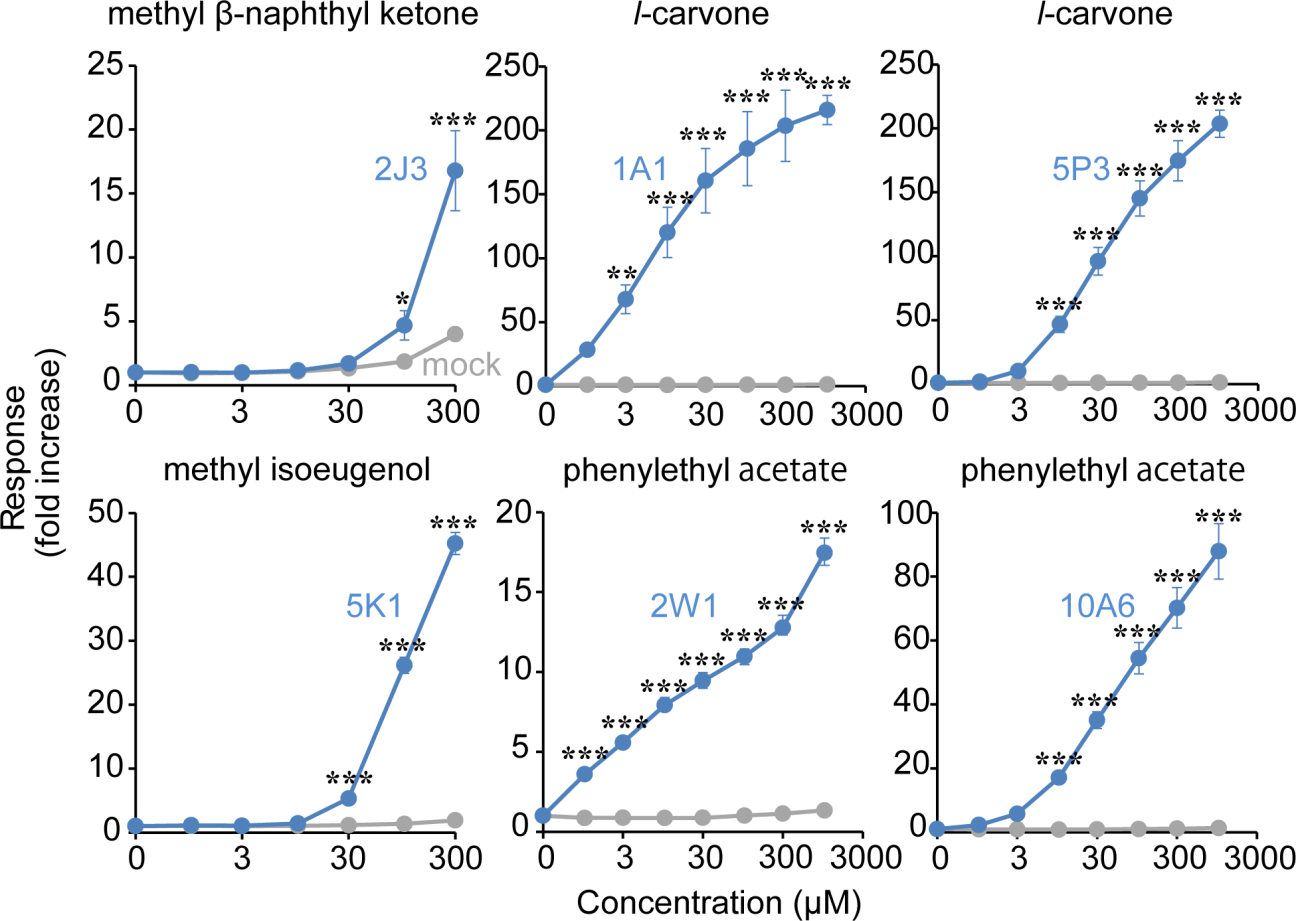
Screening new ligands of Hex-Hex Mix receptors. Dose response curves of OR1A1, OR2J3, OR2W1, OR5K1, OR5P3 and OR10A6 for indicated odorants: Methyl ß -naphthyl ketone, *l*-carvone, methyl isoeugenol, phenylethyl acetate. The HEK293 cells transfected with each OR were stimulated by 0.1 to 300 or 1000 μM of these odorants and luciferase assay was performed. Fold increases are shown as means and SE (n = 3–5). *p < 0.05, **p < 0.01, ***p < 0.001, Fisher's LSD post-test.

To screen new odorants that activated each of the Hex-Hex Mix receptors (OR1A1, OR2J3, OR2W1, OR5K1, OR5P3 and OR10A6) we stimulated ORs with 177 odorants which are commonly used in consumer products such as cosmetics, hair care products and body care products, and evaluated responses to them. Among the these odorants, methyl ß-naphthyl ketone dose-dependently activated OR2J3, *l*-carvone dose-dependently activated OR1A1 and OR5P3, methyl isoeugenol dose-dependently activated OR5K1 and phenylethyl acetate dose-dependently activated OR2W1 and OR10A6 (two-way ANOVA followed by post-hoc LSD, main effects of particular receptor versus mock transfection; OR1A1, F [1, 32] = 326.6, p < 0.001; OR2J3, F [1, 28] = 23.23, p < 0.001; OR2W1, F [1, 32] = 1577, p < 0.001; OR5K1, F [1, 28] = 1140, p < 0.001; OR5P3, F [1, 32]) = 635.6, p < 0.001; OR10A6, F [1, 32] = 479.0, p < 0.001), main effects of concentration of particular odorant; OR1A1, F [7, 32] = 18.62, p < 0.001; OR2J3, F [6, 28] = 29.85, p < 0.001; OR2W1, F [7, 32] = 99.44, p < 0.001; OR5K1, F [6, 28] = 472.9, p < 0.001; OR5P3, F [7, 32] = 75.79, p < 0.001; OR10A6, F [7, 32] = 61.05, p < 0.001), each receptor × concentration interaction; OR1A1, F [7, 32] = 18.56, p < 0.001; OR2J3, F [6, 28] = 13.89, p < 0.001; OR2W1, F [7, 32] = 90.54, p < 0.001; OR5K1, F [6, 28] = 439.1, p < 0.001; OR5P3, F [7, 32] = 75.47, p < 0.001; OR10A6, F [7, 32] = 60.02, p < 0.001) (Fig 3 and S1 Table).

**Fig 4:**
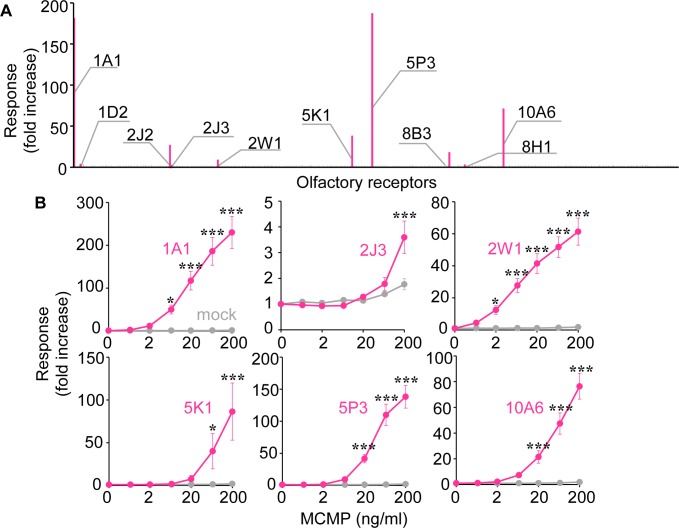
Screening of MCMP receptors from the human OR repertoire. (A) 392 human ORs were stimulated with 50 ng/ml MCMP comprised 75 μM Methyl ß -naphthyl ketone, 75 μM l-carvone, 75 μM methyl isoeugenol and 75 μM phenylethyl acetate, respectively, and luciferase assay was performed. Fold increases were calculated as mentioned in the Materials and Methods section. (B) Dose response curves of common receptors between MCMP and Hex-Hex Mix: OR1A1, OR2J3, OR2W1, OR5K1, OR5P3 and OR10A6. The HEK293 cells transfected with each OR were stimulated by 0.6 to 200 ng/ml of MCMP and luciferase assay was performed. Fold increases are shown as means and SE (n = 3–5). *p < 0.05, **p < 0.01, ***p < 0.001, Fisher's LSD post-test.

These four odorants (methyl ß-naphthyl ketone, *l*-carvone, methyl isoeugenol, and phenylethyl acetate) were combined to produce MCMP, to activate all Hex-Hex Mix receptors. While Hex-Hex Mix activated six ORs, MCMP activated 10 ORs in total: OR1A1, OR1D2, OR2J2, OR2J3, OR2W1, OR5K1, OR5P3, OR8B3, OR8H1 and OR10A6, which included all Hex-Hex Mix receptors (two-way ANOVA followed by post-hoc LSD, main effects of particular receptor versus mock transfection; [OR1A1, F [1, 42] = 115.5, p < 0.001; OR2J3, F [1, 42] = 6.780, p < 0.05; OR2W1, F [1, 42] = 206.4, p < 0.001; OR5K1, F [1, 42] = 10.89, p < 0.01; OR5P3, F [1, 42] = 135.4, p < 0.001; OR10A6, F [1, 42] = 115.2, p < 0.001), main effects of concentration of MCMP (OR1A1, F [6, 42] = 20.43, p < 0.001; OR2J3, F [6, 42] = 19.85, p < 0.001; OR2W1, F [6, 42] = 22.60, p < 0.001; OR5K1, F [6, 42] = 4.870, p < 0.001; OR5P3, F [6, 42] = 36.81, p < 0.001; OR10A6, F [6, 42 = 31.59, p < 0.001), each receptor × concentration interaction (OR1A1, F [6, 42] = 20.22, p < 0.001; OR2J3, F [6, 42] = 6.630, p < 0.001; OR2W1, F [6, 42] = 21.73, p < 0.001; OR5K1, F [6, 42] = 4.674, p = 0.001; OR5P3, F [6, 42] = 36.04, p < 0.001; OR10A6, F [6, 42] = 30.22, p < 0.001) (Fig 4, S1 Table and S1 Fig).

### Subjective scaling

**Fig 5:**
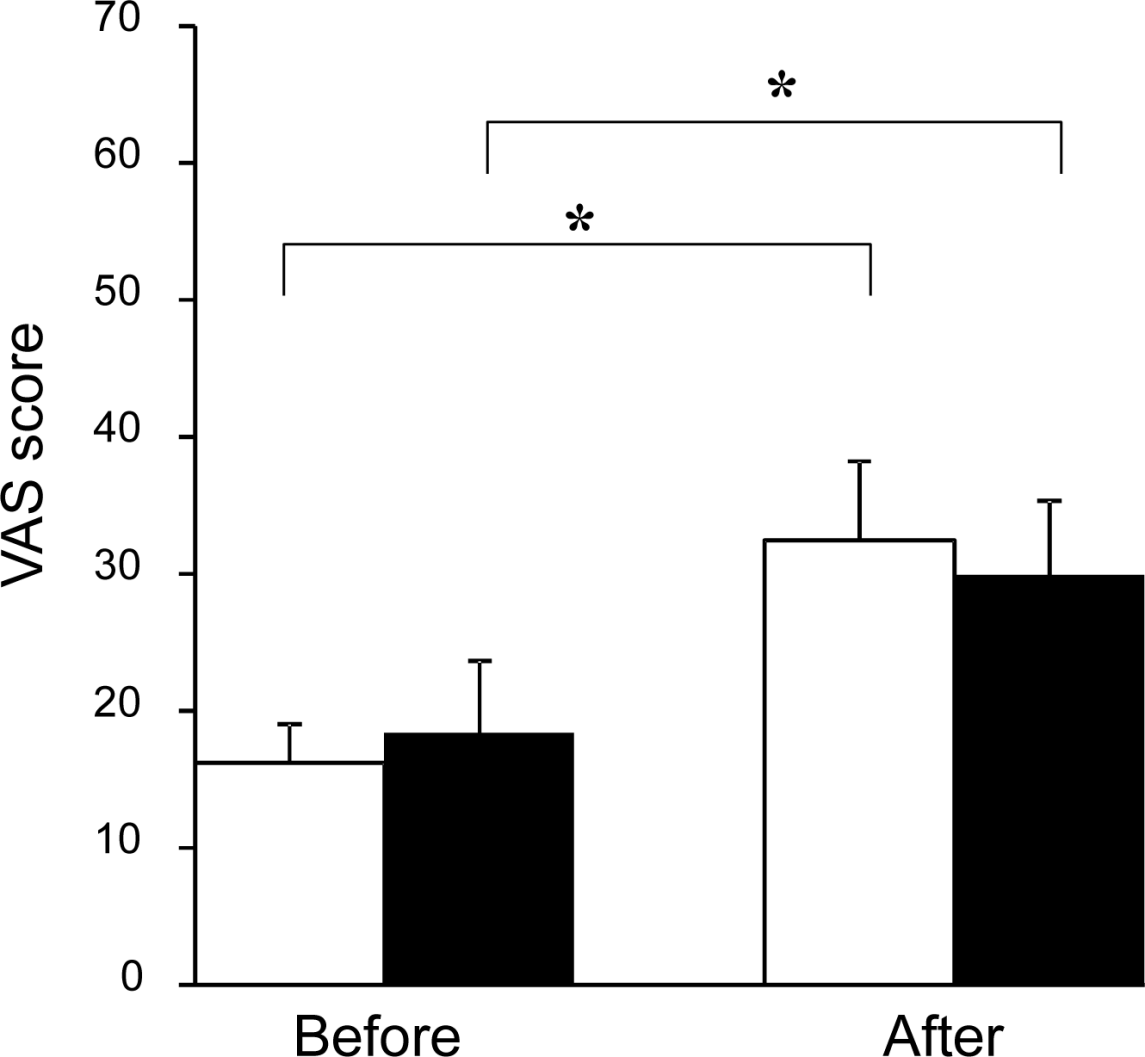
Subjective level of mental fatigue before and after the fatigue-inducing task. Open columns indicate the VAS score in the MCMP condition and closed columns indicate the VAS score in the without-MCMP condition. Data are presented as mean and SE. **p < 0.01, paired t-test with Bonferroni correction.

Subjective scaling by VAS scores for mental fatigue assessed before and after the fatigue-inducing task in the MCMP and without-MCMP conditions are shown in Fig 5. Two-way ANOVA with repeated measures was performed to compare the subjective level of mental fatigue across conditions and time points. There was a main effect of time point (F [1, 16] = 7.885, p < 0.01), but no main effect of condition or condition × time point interaction. The subjective level of mental fatigue just after the fatigue-inducing task was higher than that just before the task both in the MCMP and without-MCMP conditions (t [16] = 4.01, p = 0.001, t [16] = 4.56, p < 0.001, respectively) (p < 0.01, paired t-test with Bonferroni correction).

### Autonomic nerve activity

The Ln LF/HF ratio did not show significant changes between the values before and after the fatigue-inducing task under both conditions.

### Cognitive task

We performed two-way ANOVA with repeated measures to compare the correct response rate and reaction time of Task A and B across conditions and time points. There were no significant differences. As for Task A, there were no main effects of condition (correct response rate, F[1, 16] = 3.47, p = 0.08; reaction time, F[1, 16] = 0.55, p = 0.47), time point (correct response rate, F[1, 16] = 0.31, p = 0.59; reaction time, F[1, 16] = 1.48, p = 0.24), and condition x time point interaction (correct response rate, F[1, 16] = 1.22, p = 0.29; reaction time, F[1, 16] = 0.06, p = 0.81). As for the modified-Stroop trials of Task B, there were no main effects of condition (correct response rate, F[1, 16[ = 0.67, p = 0.43; reaction time, F[1, 16] = 1.58, p = 0.23) time point (correct response rate, F[1, 16] = 0.05, p = 0.83; reaction time, F[1, 16] = 0.05, p = 0.83), and condition x time point interaction (correct response rate, F[1, 16] = 0.02, p = 0.89; reaction time, F[1,16] = 0.03, p = 0.88). As for non-Stroop trials of Task B, there were no main effects of condition (correct response rate, F[1, 16] = 0.08, p = 0.78; reaction time, F[1, 16] = 0.47, p = 0.50), time point (correct response rate, F[1, 16] = 0.68, p = 0.42; reaction time, F[1, 16] = 0.22, p = 0.65), and condition x time point interaction (correct response rate, F[1, 16] = 0.86, p = 0.40; reaction time, F[1, 16] = 0.11, p = 0.74).

**Fig 6:**
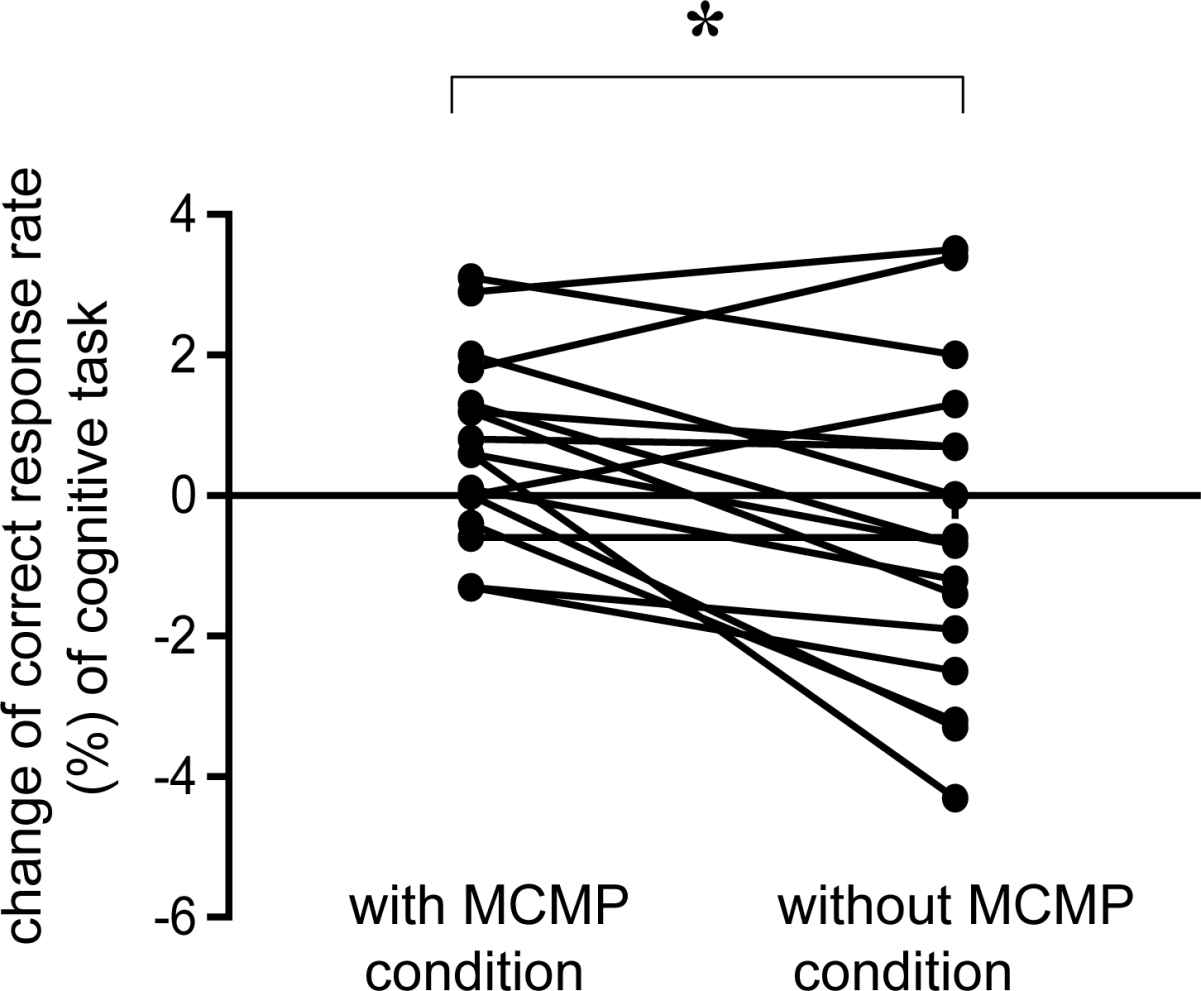
Change of correct response rate (%) in the cognitive task. Left dots indicate the correct response rate in the MCMP condition, and right dots indicate the correction rate in the without-MCMP condition. Changes in correct response rate were found in Task B for non-Stroop trials before and after the fatigue-inducing task. Data are presented as mean and SE. *p < 0.05, significant difference (paired *t*-test with Bonferroni correction).

As we focused on the correct response rate and the reaction time of Task B, we evaluated the difference values of the reaction time and correct response rate in Task B on the modified Stroop trials and non-Stroop trials before and after the fatigue-inducing task between the MCMP and the without-MCMP conditions. We performed paired t-test with the difference value of the reaction time and correct response rate between two conditions and performed paired t-tests for four times. (i.e., modified Stroop trials vs non-Stroop trials x correct response rate vs reaction time]. Therefore, we reported the p-values with Bonferroni correction based on four pairwise comparisons. We found that the difference value of the correct response rate exhibited a significant difference between the MCMP and the without-MCMP conditions (t [16] = 2.93, p < 0.05, paired t-test with Bonferroni correction). The difference value of the correct response rate in Task B on non-Stroop trials assessed before and after the fatigue-inducing task between the MCMP and without-MCMP conditions is shown in Fig 6.

## Discussion

In the present study, we developed a new odorant, MCMP, by identifying the ORs activated by Hex-Hex Mix, which has been reported to show an anti-fatigue effect [26–28]. The results revealed that the difference value of the correct response rate on non-Stroop trials in Task B in the evaluation session was significantly different between the MCMP condition and the without-MCMP condition during the fatigue-inducing session. Regarding autonomic nerve activity and subjective scaling of fatigue assessed with a VAS, no significant differences were found between the MCMP and without-MCMP conditions.

In previous studies, we reported that performance levels in Task B including simple and conflict-controlling selective attention of the modified Stroop color-word test were decreased after a fatigue-inducing session using the 2-back test [23, 33]. In addition, we showed that reaction time and correct response rate were useful as measures of performance in Task B for assessing fatigue levels to investigate possible anti-fatigue or fatigue recovery effects [23, 33]. The present results indicated that the difference value of the correct response rate of non-Stroop trials in Task B in the evaluation session was significantly different between the MCMP condition and the without-MCMP condition during the fatigue-inducing session using the 2-back test. In previous studies [23, 33], reaction time was used to assess fatigue level. In the present study, reaction time was not affected by odors. This discrepancy in the results could be caused by differences in study design between previous studies and the present study. In one previous study [23], fatigue level was assessed after a rest session following fatigue-inducing and evaluation sessions, and reaction time was found to be useful parameter. In contrast, in the present study, fatigue level was assessed just after the fatigue-inducing session, and the correct response rate was found to be useful. In another study [33], participants performed the 2-back test for 30 min, which was 10 min shorter than in the present study. These difference in the study design could have affected the different components of performance level (reaction time and correct response rate) in the assessment of the level of fatigue. Taking these previous results together, the current finding suggests that MCMP may have prevented the decrease in performance caused by the fatigue-inducing task.

The cognitive task we used for assessing performance levels before and after the fatigue-inducing session involved selective attention. Selective attention has been reported to be impaired by mental fatigue [33]. Our results suggest that MCMP could be effective for attenuating fatigue that specifically manifests as an impairment of selective attention. Previous studies reported that selective attention processes in cognitive tasks, such as modified Stroop trials, activated the prefrontal cortex (PFC) [44,45] and the anterior cingulate cortex (ACC) [46–48]. The difference value of the correct response rate in the cognitive task which requires selective attention was significantly different between the MCMP condition and the without-MCMP condition during the fatigue-inducing session. Thus, the current results suggest that MCMP may have favorable fatigue-attenuating effects in the brain region, specifically manifesting as an impairment of selective attention. In previous studies, the anti-fatigue effects of Hex-Hex Mix were proposed to operate via the autonomic system, involving the anterior cingulate gyrus [26,27]. The newly developed odorant, MCMP, derived from Hex-Hex Mix, may activate the ACC and prevent the impairment of selective attention caused by fatigue. However, considering that no significant alterations of autonomic nerve activity were found between before and after the fatigue-inducing task in the MCMP condition in the current study, the anti-fatigue effects of MCMP may involve a physiological function other than autonomic nervous system function involving the ACC.

MCMP was developed with ligands of identified Hex-Hex-Mix receptors OR1A1, OR2J3, OR2W1, OR5K1, OR5P3 and OR10A6. The current results suggest that these ORs may be related to anti-fatigue effects. Finkelmeyer et al. [49] reported that the presentation of the neutral odorant had no effect on task performance and an aversive odorant condition which induces negative emotional state was found to reduce the reaction times for incongruent stimuli of the Stroop color-word interference task only in comparison with an air control condition. The authors concluded that this difference was observed because participants experienced temporary positive emotion (i.e. ‘relief’) when stimulation with the negative olfactory stimulus was temporarily ceased during the aversive odor run. This positive emotion may then have resulted in reduced inhibition and thus increased Stroop interference. Therefore, in this previous study, it remained unclear whether the effects of the negative odor on performance were direct or indirect. In another study, Root et al. reported that cortical amygdala neurons, which were activated by attractive or aversive odors, could induce innate odor driven behavior [50]. These previous findings suggest that the activation of some specific ORs by odorants may induce specific physiological functions, emotional states and behaviors.

A previous study suggested that odorants can be transported to the bloodstream or central nervous system, directly affecting neurons in the central nervous system, where physiological conditions are controlled [51]. The present results do not provide a sufficient basis to exclude this possibility. However, another previous study reported that innate fear responses to the odor of trimethyl-thiazoline (TMT) were mediated in the dorsal domain of the olfactory bulb, and increased plasma adrenocorticotropic hormone (ACTH) levels in response to TMT-induced stress were also mediated in the dorsal domain of the olfactory bulb [6]. Thus, the current finding that two types of odorants (MCMP and Hex-Hex mix) activated the same six types of specific ORs and probably induced similar physiological functional changes, including improving performance level which may be due to the attenuation of fatigue, suggests that the peripheral olfactory system may be involved in the functional mechanisms underlying the anti-fatigue effect of odorants.

In addition, the results revealed that four other MCMP receptors (OR1D2, OR2J2, OR8B3 and OR8H1) also contributed to the difference in odor qualities between Hex-Hex Mix and MCMP. The current finding that MCMP appeared to exhibit anti-fatigue effects similar to those of Hex-Hex Mix suggests that activation of specific ORs, rather than the activation of the entire OR code that determines the odor qualities of a given odorant, could be important for inducing fatigue-attenuating effects.

The present study involved several limitations that should be considered. First, the number of participants in the present study was relatively small. Second, we did not use an odor control to examine the observed effect in more depth. Although the results suggested that MCMP had an anti-fatigue effect on performance level, the functional neural mechanisms of this effect remain unclear. Functional brain imaging studies may be useful for clarifying the neural substrates of the anti-fatigue effects of these odorants in future.

The current findings support the notion that the olfactory system is involved in inducing the anti-fatigue effects of odorants in humans. In addition, the results suggested that our newly developed odorant, MCMP, may have an anti-fatigue effect on performance level. This finding could contribute to the development of new methods for attenuating fatigue and preventing chronic fatigue, and could lead to treatments for fatigue-related diseases.

## Supporting information

S1 Fig MCMP receptors screening from the human OR repertoire. Dose response curves of OR1D2, OR2J2, OR8B3 and OR8H1 for MCMP. HEK293 cells transfected with each OR were stimulated with 0.6 to 200 ng/ml of MCMP and the luciferase assay was performed. Fold increases are shown as means and SE (n = 3–5). Two-way ANOVA followed by post-hoc LSD revealed significant main effects of particular receptor versus mock transfection [OR1D2, F [1, 28] = 26.88, p < 0.001; OR2J2, F [1, 28] = 58.96, p < 0.001; OR8B3, F [1, 28] = 21.65, p < 0.001; OR8H1, F [1, 28] = 20.11, p < 0.001], main effects of concentration of MCMP [OR1D2, F [6, 28] = 29.58, p < 0.001; OR2J2, F [6, 28] = 15.4, p < 0.001; OR8B3, F [6, 28] = 10.84, p < 0.001; OR8H1, F [6, 28] = 37.17, p < 0.001] and each receptor × concentration interaction [OR1D2, F [6, 28] = 16.69, p < 0.001; OR2J2, F [6, 28] = 14.10, p < 0.001; OR8B3, F [6, 28] = 9.633, p < 0.001; OR8H1, F [6, 28] = 13.57, p < 0.001], *p < 0.05, **p < 0.01, ***p < 0.001, Fisher's LSD post-test.
(TIF)

S1 Table Results of two-way analyses of variance of candidate receptors. (DOCX)
