# Supplementary figures and images for: Involvement of the olfactory system in the induction of anti-fatigue effects by odorants
Source: PLoS One. 2018 Mar 29;13(3):e0195263. doi: 10.1371/journal.pone.0195263 (PMC5875884; doi:10.1371/journal.pone.0195263)

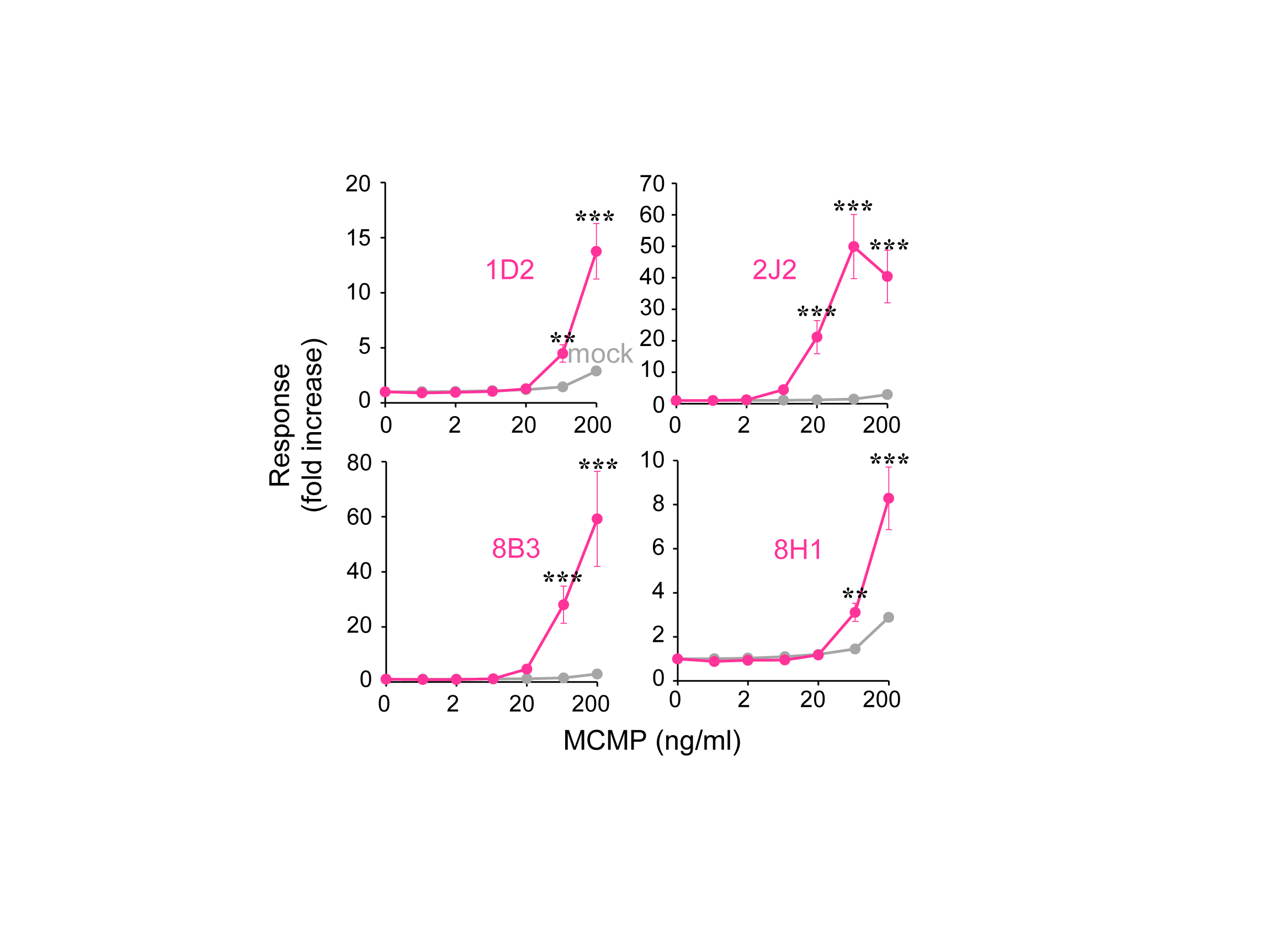

Supplement: S1 Fig — Dose response curves of OR1D2, OR2J2, OR8B3 and OR8H1 for MCMP. HEK293 cells transfected with each OR were stimulated with 0.6 to 200 ng/ml of MCMP and the luciferase assay was performed. Fold increases are shown as means and SE (n = 3–5). Two-way ANOVA followed by post-hoc LSD revealed significant main effects of particular receptor versus mock transfection [OR1D2, F [1, 28] = 26.88, p < 0.001; OR2J2, F [1, 28] = 58.96, p < 0.001; OR8B3, F [1, 28] = 21.65, p < 0.001; OR8H1, F [1, 28] = 20.11, p < 0.001], main effects of concentration of MCMP [OR1D2, F [6, 28] = 29.58, p < 0.001; OR2J2, F [6, 28] = 15.4, p < 0.001; OR8B3, F [6, 28] = 10.84, p < 0.001; OR8H1, F [6, 28] = 37.17, p < 0.001] and each receptor × concentration interaction [OR1D2, F [6, 28] = 16.69, p < 0.001; OR2J2, F [6, 28] = 14.10, p < 0.001; OR8B3, F [6, 28] = 9.633, p < 0.001; OR8H1, F [6, 28] = 13.57, p < 0.001], *p < 0.05, **p < 0.01, ***p < 0.001, Fisher's LSD post-test. (TIF) [file pone.0195263.s001.tif]
