# Supplementary material for: Involvement of the olfactory system in the induction of anti-fatigue effects by odorants
Source: PLoS One. 2018 Mar 29;13(3):e0195263. doi: 10.1371/journal.pone.0195263 (PMC5875884; doi:10.1371/journal.pone.0195263)
Supplement: S1 Table — (DOCX) [file pone.0195263.s002.docx]

|  | | Interaction | | Concentration of odorant | | Particular receptor  versus mock transfection | |
| --- | --- | --- | --- | --- | --- | --- | --- |
| Odorant | Receptor | F (DFn, DFd) | P value | F (DFn, DFd) | P value | F (DFn, DFd) | P value |
| Hex-Hex Mix | OR1A1 | F (4, 50) = 5.341 | P = 0.0012 | F (4, 50) = 30.40 | P < 0.0001 | F (1, 50) = 11.73 | P = 0.0012 |
| Hex-Hex Mix | OR2J3 | F (4, 40) = 4.727 | P = 0.0032 | F (4, 40) = 8.259 | P < 0.0001 | F (1, 40) = 6.294 | P = 0.0163 |
| Hex-Hex Mix | OR2W1 | F (4, 20) = 4.078 | P = 0.0141 | F (4, 20) = 5.419 | P = 0.0040 | F (1, 20) = 6.541 | P = 0.0188 |
| Hex-Hex Mix | OR4D9 | F (4, 50) = 0.2264 | P = 0.9224 | F (4, 50) = 21.92 | P < 0.0001 | F (1, 50) = 2.987 | P = 0.0901 |
| Hex-Hex Mix | OR5K1 | F (4, 20) = 4.893 | P = 0.0065 | F (4, 20) = 22.29 | P < 0.0001 | F (1, 20) = 4.900 | P = 0.0386 |
| Hex-Hex Mix | OR5P3 | F (4, 20) = 24.57 | P < 0.0001 | F (4, 20) = 35.29 | P < 0.0001 | F (1, 20) = 28.20 | P < 0.0001 |
| Hex-Hex Mix | OR10A6 | F (4, 40) = 4.836 | P = 0.0028 | F (4, 40) = 17.24 | P < 0.0001 | F (1, 40) = 9.141 | P = 0.0043 |
| *l*-carvone | OR1A1 | F (7, 32) = 18.56 | P < 0.0001 | F (7, 32) = 18.62 | P < 0.0001 | F (1, 32) = 326.6 | P < 0.0001 |
| methyl ß-naphthyl ketone | OR2J3 | F (6, 28) = 13.89 | P < 0.0001 | F (6, 28) = 29.85 | P < 0.0001 | F (1, 28) = 23.23 | P < 0.0001 |
| phenylethyl acetate | OR2W1 | F (7, 32) = 90.54 | P < 0.0001 | F (7, 32) = 99.44 | P < 0.0001 | F (1, 32) = 1577 | P < 0.0001 |
| methyl isoeugenol | OR5K1 | F (6, 28) = 439.1 | P < 0.0001 | F (6, 28) = 472.9 | P < 0.0001 | F (1, 28) = 1140 | P < 0.0001 |
| *l*-carvone | OR5P3 | F (7, 32) = 75.47 | P < 0.0001 | F (7, 32) = 75.79 | P < 0.0001 | F (1, 32) = 635.6 | P < 0.0001 |
| phenylethyl acetate | OR10A6 | F (7, 32) = 60.02 | P < 0.0001 | F (7, 32) = 61.05 | P < 0.0001 | F (1, 32) = 479.0 | P < 0.0001 |
| MCMP | OR1A1 | F (6, 42) = 20.22 | P < 0.0001 | F (6, 42) = 20.43 | P < 0.0001 | F (1, 42) = 115.5 | P < 0.0001 |
| MCMP | OR1D2 | F (6, 28) = 16.69 | P < 0.0001 | F (6, 28) = 29.58 | P < 0.0001 | F (1, 28) = 26.88 | P < 0.0001 |
| MCMP | OR1D5 | F (6, 28) = 0.3188 | P = 0.9216 | F (6, 28) = 30.87 | P < 0.0001 | F (1, 28) = 0.07780 | P = 0.7824 |
| MCMP | OR2J2 | F (6, 28) = 14.10 | P < 0.0001 | F (6, 28) = 15.43 | P < 0.0001 | F (1, 28) = 58.96 | P < 0.0001 |
| MCMP | OR2J3 | F (6, 42) = 6.630 | P < 0.0001 | F (6, 42) = 19.85 | P < 0.0001 | F (1, 42) = 6.780 | P = 0.0127 |
| MCMP | OR2W1 | F (6, 42) = 21.73 | P < 0.0001 | F (6, 42) = 22.60 | P < 0.0001 | F (1, 42) = 206.4 | P < 0.0001 |
| MCMP | OR5K1 | F (6, 42) = 4.674 | P = 0.0010 | F (6, 42) = 4.870 | P = 0.0007 | F (1, 42) = 10.89 | P = 0.0020 |
| MCMP | OR5P3 | F (6, 42) = 36.04 | P < 0.0001 | F (6, 42) = 36.81 | P < 0.0001 | F (1, 42) = 135.4 | P < 0.0001 |
| MCMP | OR8B3 | F (6, 28) = 9.633 | P < 0.0001 | F (6, 28) = 10.84 | P < 0.0001 | F (1, 28) = 21.65 | P < 0.0001 |
| MCMP | OR8H1 | F (6, 28) = 13.57 | P < 0.0001 | F (6, 28) = 37.17 | P < 0.0001 | F (1, 28) = 20.11 | P = 0.0001 |
| MCMP | OR10A6 | F (6, 42) = 30.22 | P < 0.0001 | F (6, 42) = 31.59 | P < 0.0001 | F (1, 42) = 115.2 | P < 0.0001 |
| MCMP | OR10C1 | F (6, 28) = 1.106 | P = 0.3837 | F (6, 28) = 33.42 | P < 0.0001 | F (1, 28) = 1.462 | P = 0.2368 |
| MCMP | OR10K1 | F (6, 28) = 0.3627 | P = 0.8962 | F (6, 28) = 32.59 | P < 0.0001 | F (1, 28) = 0.07481 | P = 0.7865 |
| MCMP | OR51F2 | F (6, 28) = 2.048 | P = 0.0922 | F (6, 28) = 24.35 | P < 0.0001 | F (1, 28) = 9.493 | P = 0.0046 |
